# Epigenome-wide association study meta-analysis of wellbeing

**DOI:** 10.1186/s13148-026-02202-0

**Published:** 2026-07-14

**Authors:** Meike Bartels, Margot P. van de Weijer, Natalia Azcona-Granada, Bart M. L. Baselmans, Matthew Suderman, Mette Soerensen, Jadwiga Buchwald, Rosa H. Mulder, Rajesh Rawal, Michelle Luciano, Marc Jan Bonder, Priyanka Choudhary, Estelle Lowry, Penelope A. Lind, Joel Schwartz, Birgit Debrabant, Miina Ollikainen, Janine F. Felix, Marian J. Bakermans-Kranenburg, Henning Tiemeier, Christian Gieger, Melanie Waldenberger, Nicholas G. Martin, Pantel Vokonas, Andrea A. Baccarelli, Kaare Christensen, Jaakko Kaprio, Marinus H. van IJzendoorn, Rebecca T. Emeny, Ian J. Deary, Lude Franke, Sylvain Sebert, Allan F. McRae, Avron Spiro, Jenny van Dongen

**Affiliations:** 1 Department of Biological Psychology, Vrije Universiteit Amsterdam, Van Der Boechorststraat 7, 1081 BT Amsterdam, The Netherlands; 2 Amsterdam Public Health Research Institute, Amsterdam University Medical Centre, Amsterdam, The Netherlands; 3 Department of Psychiatry, Location AMC, Amsterdam University Medical Centre, Amsterdam, The Netherlands; 4 MRC Integrative Epidemiology Unit, Bristol Medical School, Population Health Sciences, University of Bristol, Bristol, UK; 5 The Danish Twin Registry and the Research Unit for Epidemiology, Biostatistics and Biodemography, Department of Public Health, University of Southern Denmark, Odense, Denmark; 6 Department of Clinical Genetics, Odense University Hospital, Odense, Denmark; 7 Institute for Molecular Medicine Finland FIMM, University of Helsinki, Helsinki, Finland; 8 The Generation R Study Group, Erasmus MC, University Medical Center Rotterdam, Rotterdam, the Netherlands; 9 Department of Child and Adolescent Psychiatry/Psychology, Erasmus MC, University Medical Center Rotterdam, Rotterdam, the Netherlands; 10 Department of Microbiology, Laboratoire National de Santé, Dudelange, Luxembourg; 11 Department of Psychology, University of Edinburgh, Edinburgh, UK; 12 Department of Genetics, University of Groningen, University Medical Centre Groningen, Groningen, The Netherlands; 13 Oncode Institute, Utrecht, The Netherlands; 14 Research Unit of Population Health, Faculty of Medicine, University of Oulu, Oulu, Finland; 15 Queen’s University Belfast, Belfast, UK; 16 Psychiatric Genetics, Brain and Mental Health Research Program, QIMR Berghofer, Brisbane, Australia; 17 School of Biomedical Sciences, Queensland University of Technology, Brisbane, Australia; 18 School of Biomedical Sciences, University of Queensland, Brisbane, Australia; 19 Department of Environmental Health, Harvard School of Public Health, Boston, USA; 20 Data Science and Statistics, Department of Mathematics and Computer Science, University of Southern Denmark, Odense, Denmark; 21 Minerva Foundation Institute for Medical Research, Helsinki, Finland; 22 Department of Pediatrics, Erasmus MC, University Medical Center Rotterdam, Rotterdam, the Netherlands; 23 William James Center for Research, ISPA Instituto Universitário, Lisbon, Portugal; 24 Faculty of Psychology and Humanities, Universidad San Sebastián, Valdivia, Chile; 25 Department of Social and Behavioral Science, Harvard T.H. Chan School of Public Health, Boston, USA; 26 Research Unit Molecular Epidemiology, Institute of Epidemiology, Helmholtz Zentrum München, German Research Center for Environmental Health, Neuherberg, Germany; 27 Genetic Epidemiology, Brain and Mental Health Research Program, QIMR Berghofer, Brisbane, Australia; 28 Veterans Affairs Boston Healthcare System, Boston, USA; 29 Harvard T.H. Chan School of Public Health, Boston, USA; 30 Psychiatry Monash Health, Monash University, Melbourne, Australia; 31 Department of Internal Medicine, Division of Molecular Medicine, UNM Comprehensive Cancer Center, Cancer Control & Population Sciences Research Program, Albuquerque, USA; 32 Lothian Birth Cohorts, Psychology, University of Edinburgh, Edinburgh, UK; 33 Institute for Molecular Bioscience, The University of Queensland, Brisbane, Australia; 34 Amsterdam Reproduction & Development (AR&D) Research Institute, Amsterdam, The Netherlands

## Abstract

Wellbeing is associated with both behavioral phenotypes as well as several key life outcomes, such as health, employment, and coping with stressful events. These phenotypes associated with wellbeing could be potential indicators of differential epigenetic patterns between individuals that differ in their levels of wellbeing. We performed the largest epigenome-wide (EWAS) meta-analysis of wellbeing to date by combining whole blood DNA methylation data (Illumina 450K array) from 13 cohorts from Europe, Australia, and the USA (*N* = 10,757 participants). After correcting for smoking and BMI, no epigenome-wide significant methylation sites were identified. We tested whether a weighted methylation score (MS) based on leave-one-cohort-out EWAS meta-analysis summary statistics predicted wellbeing in an independent cohort, and whether prediction was significant over and above the polygenic score (PGS) for wellbeing. The MS was associated with wellbeing (variance explained = 0.22%, *p* = 0.03) and was no longer significant after adding the polygenic score (PGS; variance explained = 0.43%, *p* = 0.0046, MS; variance explained = 0.07%, *p* = 0.2842). We further compared DNA methylation levels in 16 pairs of monozygotic twins discordant for wellbeing. These analyses revealed no significant within-pair DNA methylation differences at the top-sites from the meta-analysis or in MS. Our results suggest that larger EWAS meta-analyses with uniform phenotype assessment are required to identify methylation sites associated with wellbeing.

**Supplementary Information:**

The online version contains supplementary material available at https://doi.org/10.1186/s13148-026-02202-0.

## Introduction

There has been progress in the identification of genetic variants influencing individual differences in wellbeing [1–4]. For example, the most recent meta-analysis by Baselmans and colleagues identified over 300 loci jointly explaining about 1% of the phenotypic variation [4]. The next challenge is to improve our understanding of the biological effects of these genetic susceptibility loci, especially since the actual causal genes are not necessarily proximal to the lead SNPs identified in genome-wide association studies (GWASs). Supported by the observation that GWAS variants are preferentially located in enhancers and open chromatin regions [5, 6], the majority of common genetic risk factors are predicted to influence gene regulation through effects on epigenetic marks such as DNA methylation rather than directly affecting the coding sequence of transcribed proteins [7]. As a GWAS follow-up analysis, we previously integrated information from methylation Quantitative Trait Loci (mQTLs; SNPs associated with DNA methylation) and GWAS summary statistics of wellbeing to identify SNPs that affect wellbeing via DNA methylation [4]. This approach, which is named MWAS (Methylome-Wide Association Study), identified 913 CpGs whose methylation level mediated the effect of *cis* genetic variants on wellbeing [4].

Accumulating lines of evidence point towards a key role for epigenetic mechanisms regulating gene expression during brain development, maturation, and learning [8]. Besides playing an important role in the development of the brain, DNA methylation has been linked to various health outcomes that are associated with wellbeing, such as obesity [9], type 2 diabetes [10, 11], and depression [12, 13]. Variation in DNA methylation between individuals can be the result of environmental influences [14–16]. For instance, various early life exposures such as childhood abuse [17], and prenatal exposure to maternal famine [18] can induce long-term changes in DNA methylation [17–19]. Additionally, methylation variation can result from exposures later in life. Epigenome-wide association studies (EWASs) try to identify epigenetic (usually DNA methylation) variation linked to diseases, traits, or environmental exposures. Cigarette smoking is perhaps the best studied example in human EWASs and has been associated with differential methylation at thousands of CpG sites [20, 21].

Wellbeing is linked to numerous exposures and behaviors across the life course, such as income and employment, health, neighborhood environment (e.g., neighborhood safety), smoking, stress, alcohol use, and several social factors such as friendship patterns [22]. These exposures and behavioral characteristics are potential candidate drivers of differential epigenetic patterns between individuals reporting higher or lower levels of wellbeing. We previously investigated the association between DNA methylation in blood and wellbeing in the Netherlands Twin Register (NTR, *N* = 2519) using an EWAS approach, reporting two CpG sites that showed a statistically significant association after Bonferroni correction [23]. Gene ontology (GO) analysis highlighted enrichment of several central nervous system categories among methylation sites with the smallest *p*-values. However, replication of these results in larger samples is warranted.

Here, we performed a large (*N* = 10,757) EWAS meta-analysis of whole blood DNA methylation data (Illumina 450K array) from 13 cohorts with various measures of wellbeing. In the NTR cohort, we subsequently assessed the predictive value of wellbeing DNA methylation scores (MS) created using weights from a leave-one-out meta-analysis without NTR. We also tested if the MS predicted wellbeing over and above the polygenic score (PGS) for wellbeing, to examine if PGS and MS capture independent information about wellbeing. Finally, in a unique group of monozygotic (MZ) twins from pairs discordant for wellbeing, we exploratively tested for differentially methylated CpG sites, ruling out possible confounding genetic -and environmental factors.

## Methods

### Epigenome-wide association study

Data on wellbeing, white blood cell counts, and DNA methylation levels were available for 13 cohorts (*N* = 10,757) of which 10 cohorts (*N* = 8,675) also had information on the covariates body mass index (BMI) and smoking status (see Table 1). The included cohorts were from Europe, Australia, and the United States (European ancestry). All participants provided written informed consent regarding participation in individual studies, and all studies were approved by the local research ethics committees or institutional review boards. More information on the individual cohorts can be found in Supplementary Tables 1.

**Table 1:** Cohort characteristics

Cohort	N M1	N M2	% Female	% Current smoker	% Former smoker	Mean DNA age^a^ (SD)	Sample type
ALSPAC	826	826	100	27%	14%	47.5 (4.4)	Whole blood
DTR	1012	1012	52%	23%	36%	64.2 (14.4)	Whole blood
FTC	593	591	57%	46%*		22.5 (0.73)	Whole blood
GenR	660	–	NA	NA	NA	0 (0)	Cord blood
KORA	660	660	50%	6%	45%	71 (4)	Whole blood
LBC1921	378	376	59%	7%	47%	79.3 (0.6)	Whole blood
LBC1936	697	697	48%	9%	42%	72.5 (0.7)	Whole blood
LLD	730	730	59%	47%	34%	45.6 (13)	Whole blood
NFBC66	740	740	57%	26%	20%	31 (0)	Whole blood
NFBC86	524	524	54%	20%	37%	16 (0)	Whole blood
NTR	2519	2506	56%	15%	18%	36.8 (5)	Whole blood
QIMR	233	–	55%	–	–	14.7 (2.4)	Whole blood
NAS	1185	–	0%	3%	63%	75.3 (6.8)	Whole blood

M1, Model 1: correction for sex, age at blood sampling, white blood cell percentages (measured or imputed), and technical covariates; M2, Model 2 included the same predictors plus body-mass-index (BMI) and smoking status in adolescents and adults (current smoker, former smoker or never smoked); ALSPAC, Avon longitudinal study of parents and children; DTR, Danish twin registry; FTC, Finish twin cohort; GenR, Generation R; KORA, Cooperative health research in Augsburg Region; LBC1921, Lothian Birth Cohort 1921; LBC1936, Lothian birth cohort 1936; LLD, Lifelines-deep; NFBC66, Northern Finland birth cohort 1966; NFBC86, Northern Finland birth cohort 1986; NTR, Netherlands twin register; QIMR, Brisbane longitudinal twin study; NAS, Normative aging study

^*^FTC only had 2 categories for smoking: non-smoking or ever-smoked. The percentage thus shows current and former smokers combined

^a^Age at DNA sample collection

### Wellbeing measurements

Detailed information on the wellbeing measures for each cohort are provided in Supplementary Table 2. All items (single or summed) were wellbeing questions, except for the measure used inLifelines (LLD). LLD derived a sumscore from the positive-affect negative-affect (PANAS) questionnaire [24] with items such as ‘interested’, ‘enthusiastic’, ‘proud’ and ‘inspired’.

### DNA methylation BeadChips

DNA methylation was assessed with the Illumina HumanMethylation450 BeadChip (450K array) in whole or cord blood. All cohorts analyzed DNA methylation β-values, which range from 0 to 1, indicating the proportion of DNA that is methylated at a CpG in a sample. More information on the methylation data of the individual cohorts is presented in Supplementary Table 3.

### Epigenome-wide association analysis

EWAS analyses were performed according to a standard operating procedure (See Supplementary Information 2). In each cohort, the association between DNA methylation level and wellbeing was tested under a linear model with DNA methylation at one CpG site as outcome, and correction for relatedness of individuals, using the r package generalized estimation equations (gee), where applicable (see Supplementary Table 4). Gee corrects for familial relatedness by using an exchangeable conditional covariance matrix and tests are based on robust (sandwich corrected) standard errors. Two models were fitted. The first model included the following predictors: wellbeing, sex (if applicable), age at blood sampling (if applicable), the square of the Z-score of age at blood sampling (hereafter referred to as standardized (age at blood sampling)^2^, white blood cell percentages (measured or imputed), and cohort-specific technical covariates (such as sample plate and array row). The second model additionally included BMI and smoking status (current smoker/former smoker/never smoked or ever smoker/never smoker). We adjusted for BMI and smoking because they are known to have large effects on DNA methylation and can be confounders [20, 25].

### Quality control

The following methylation sites were removed from the meta-analysis: methylation sites on the sex chromosomes, methylation sites with more than 5% missing data in a cohort, methylation sites interrogated by probes overlapping with SNPs with a minor allele frequency (MAF) > 0.01 in the 1000G EU population or GONL population that affect the CpG or single base extension site [26], and ambiguously mapping probes reported by Chen et al. with an overlap of at least 47 bases per probe [27]. In addition, we applied a minimum sample size filter (N > 10,000 for model 1), to remove probes that were missing in multiple cohorts. The number of probes after filtering was 387,188. Detailed cohort-level quality control and filtering of the methylation data is described in Supplementary Table 3. The R package Bacon was used to compute the Bayesian inflation factor and to obtain bias- and inflation-corrected test statistics prior to meta-analysis [28].

### Meta-analysis

Fixed-effects meta-analyses were performed in METAL [29]. We used the *p*-value-based (sample size-weighted) method because the measurement scale of wellbeing measures differs across studies. The I^2^ statistic from METAL was used to describe between-study heterogeneity in CpG-specific effect size estimates across cohorts included in the meta-analysis. Statistical significance was assessed considering Bonferroni correction for the number of sites tested (α = 1.3 × 10^–7^). Additionally, we considered the False Discovery Rate (5%). False discovery rate (FDR) q-values were computed with the R package q-value with default settings.

### Methylation score

Based on summary statistics from the whole blood EWAS meta-analysis of wellbeing, DNA methylation scores (MS) were created in the adult NTR cohort (whole blood 450k array data) to test the combined predictive value of DNA methylation sites associated with wellbeing in whole blood [30, 31]. For this purpose, the meta-analysis of EWAS model 2 was rerun after excluding NTR (new *N* = 6156). We applied a minimum sample size filter (N > 6000 for model 2) to remove probes with high missingness. For each NTR participant, a weighted sum score was calculated by multiplying the methylation value at a given CpG by the effect size (z-score) from the model 2 EWAS meta-analysis, and then summing these values over all genome-wide CpGs:

$$ MS = Z_{1} \times CpG_{1} + Z_{2} \times CpG_{2} ... + Z_{i} \times CpG_{i} $$

where MS = Methylation score, CpG_i_ is the methylation level at CpG site i in the NTR participant, and Z_i_ is the effect size (z-score) at CpGi from the EWAS meta-analysis without NTR. We compared the performance of DNA methylation scores generated with pruning (removing CpGs with a correlation > 0.1) or without pruning, as previously described in Odintsova et al. 2021 [31], and compared scores generated with various subsets of CpGs based on different meta-analysis p-value thresholds: *p* < 1 (all CpGs), *p* < 1 × 10^−1^, *p* < 1 × 10^−2^, *p* < 1 × 10^−3^, *p* < 1 × 10^−4^ and *p* < 1 × 10^−5^. Pruning is performed to remove correlated CpGs that are redundant (and potentially add noise to scores). The expectation is that if the set of CpGs associated with a trait is correlated (and especially if correlations are strong or abundant), pruning will improve performance of the MS.

Wellbeing scores were regressed on the MSs with gee models, which were fitted with the R package ‘gee’. The following settings were used: Gaussian link function (for continuous data), 100 iterations, and the ‘exchangeable’ option to account for the correlation structure within families and within persons. Wellbeing was regressed on each MS plus the same covariates as included in EWAS model 2; white blood cell counts, sample plate (dummy-coded), array row, age at blood sampling, standardized (age at blood sampling)^2^, sex, BMI, and smoking (current smoker/former smoker /never smoked) as covariates. In each analysis, MSs and wellbeing scores were standardized, that is, the mean was subtracted from each score and the remaining value was then divided by the standard deviation. This makes the estimated regression coefficient for the effect of the predictor MS on the outcome wellbeing equivalent to a (semipartial) correlation. The proportion of variance in wellbeing explained by the MSs, after accounting for covariates, can subsequently be obtained by squaring the regression coefficient. This value was multiplied by 100 to obtain the percentage of variance explained.

### Combined analysis of a polygenic score and methylation score

Next, we took the best-performing MS and tested whether the MS significantly predicted wellbeing over and above a polygenic score (PGS) based on GWAS summary statistics. For this purpose, we used the N-weighted genome-wide association meta-analysis (NGWAMA) from Baselmans et al. [4] excluding samples from the Netherlands Twin Register. Using these summary statistics, polygenic scores were created using the following strategy.

The weights used for the polygenic scores were based on the wellbeing spectrum multivariate NGWAMA including the highly genetically correlated measures of life satisfaction, positive affect, depression, and neuroticism. Quality control parameters for genotype data in NTR are described in Supplementary Table 5. In total, 1,224,793 SNPs passed QC and were used to construct polygenic scores. Before aggregating the individual effect sizes into polygenic scores using plink [32], we adjusted the effect sizes for linkage disequilibrium (LD) using LDpred [33]. LDpred requires specifying the fraction of SNPs with a non-zero effect on wellbeing, which was set to 1. The prediction of wellbeing by PGS and MS was tested with the R package ‘gee’. We corrected for the same covariates as in the EWAS (that is: age at blood sampling, standardized (age at blood sampling)^2^, sex, BMI, smoking (current smoker/former smoker /never smoked), sample plate (dummy-coded), array row, and percentages of monocytes, eosinophils, and neutrophils), plus the first 10 genomic principal components (PCs), and genotyping platform dummies.

### Discordant twin pair analysis

To control for genetic and shared environmental confounding we used the unique characteristics of monozygotic (MZ) twin pairs to help understand the association between DNA methylation and wellbeing. MZ co-twins share nearly 100% of their DNA sequence as well as their common environment during childhood and adolescence. Therefore, MZ twins from pairs discordant for wellbeing form the ideal case–control groups because they are perfectly matched on many possible confounding factors, including age, sex, genetic make-up, and numerous shared environmental factors [34].

**Fig. 1:**
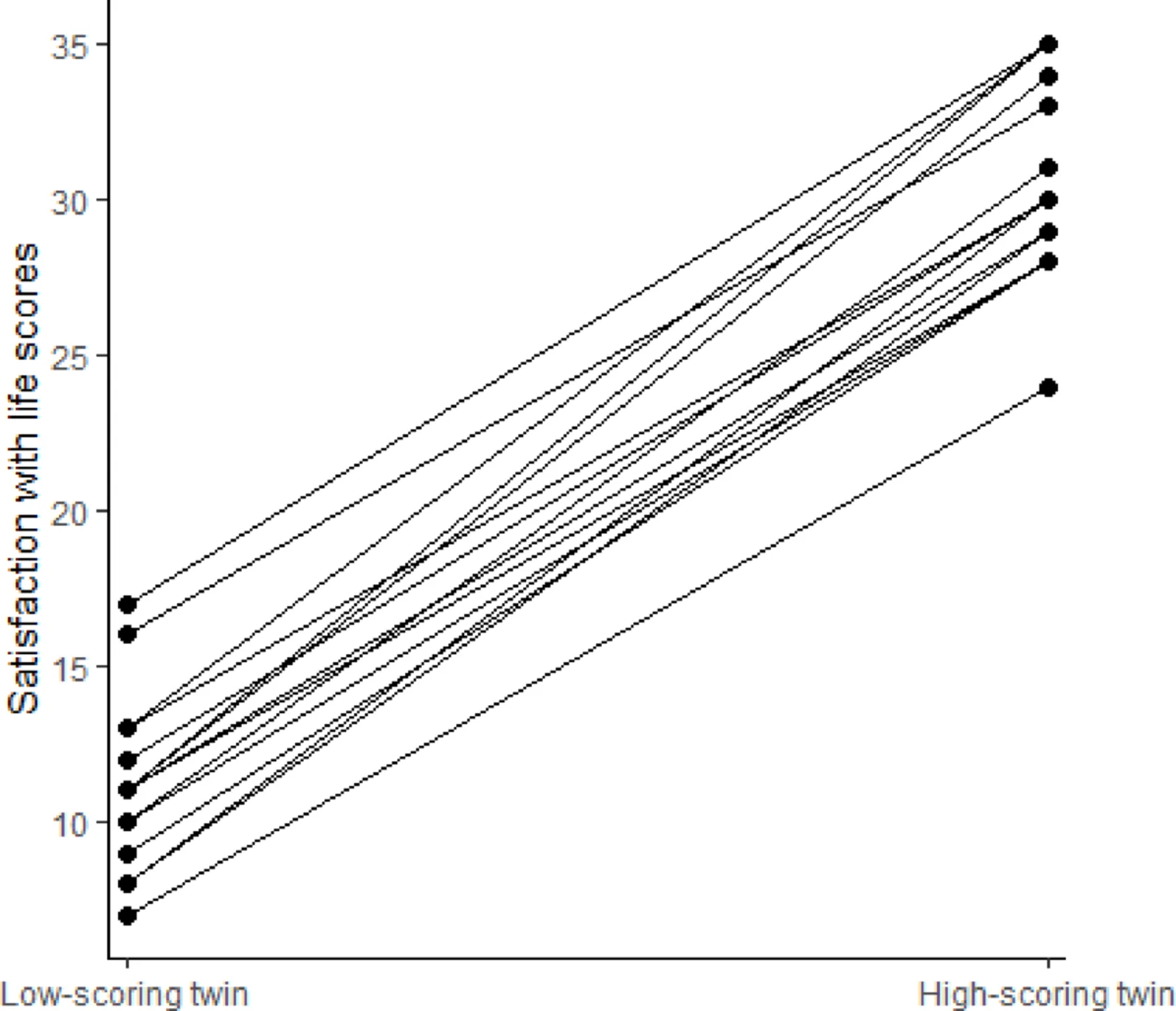
Satisfaction with life scores for monozygotic twin pairs discordant for wellbeing (N = 16)

As the criterion for discordance, we selected twins who had a within-pair difference wellbeing score of > 3 standard deviations (SDs) on the satisfaction with life scale. Based on this threshold, 16 MZ twin pairs out of 624 MZ twin pairs (2%) from the NTR with data on wellbeing and DNA methylation were classified as discordant for wellbeing. The mean wellbeing score for the high-scoring twin (Mean_wellbeing_ = 30.62) was on average 19 points higher than that of the low-scoring twin (Mean_wellbeing_ = 11.06) (see Fig. 1), while the mean (SD) of the full sample was 27.07 (5.46) (See Table 2). Additionally, we identified twins from pairs concordant in their wellbeing score, i.e., with identical wellbeing scores (N = 91). From these concordant twin pairs, we randomly selected 16 concordant twin pairs (Mean_wellbeing_ = 29) to correspond to the number of discordant twin pairs. The data from concordant pairs were used to compare the distribution of within-pair DNA methylation differences.

**Table 2:** Descriptives wellbeing (WB) discordant MZ twins

	Low WB twin	High WB twin
Mean WB (SD)	11.06 (2.72)	30.63 (3.11)
WB range	7–17	24–35
*N* males/females	1/15	1/15
Mean age (SD)^a^	36.51(11.59)	36.36 (11.69)
Mean BMI (SD)	24.31 (5.15)	24.96 (5.15)
Neutrophil (%)	51.18 (9.34)	53.34 (10.26)
Monocyte (%)	7.86 (1.81)	8.05 (2.24)
Eosinophil (%)	2.74 (1.67)	3.03 (2.24)
*N* non-smoker/former smoker/current smoker	13/3/0	11/4/1

WB, Wellbeing. ^a^Age at DNA sample collection. Note: in the Netherlands Twin Register, participants were visited at home for collection of blood samples. Minor differences in age at sample collection reflect that co-twins could not always be visited on the same day

We compared the similarity in methylation levels in discordant MZ pairs and concordant MZ twin pairs. DNA methylation levels were residualized for BMI, smoking, sample plate (dummy coded), array row, and percentages of monocytes, eosinophils, and neutrophils in advance with a linear regression model (i.e., the same covariates as included in the EWAS minus age and sex because MZ twins have the same age and sex and the blood sample was collected at the same age). The residuals were used as input for paired t-tests. We tested whether the top 10 CpG sites with the lowest *p*-value from the leave-one-out EWAS meta-analysis (model 2) excluding NTR showed differences in DNA methylation in the discordant MZ pairs at a Bonferroni-corrected significance threshold of α = 0.05/10 = 0.005, and tested if the overall distribution of the average absolute within-pair differences of residualized methylation beta-values differed between discordant and concordant twin pairs for the top 10 CpGs using a Kolmogorov–Smirnov test. Second, we tested if the DNA methylation score for wellbeing differed in discordant MZ pairs with a paired t-test. For this, we took the best-performing DNA methylation score from the DNA methylation score analysis (the pruned score based on all genome-wide CpGs).

## Results

### Peripheral blood meta-analyses

**Fig. 2:**
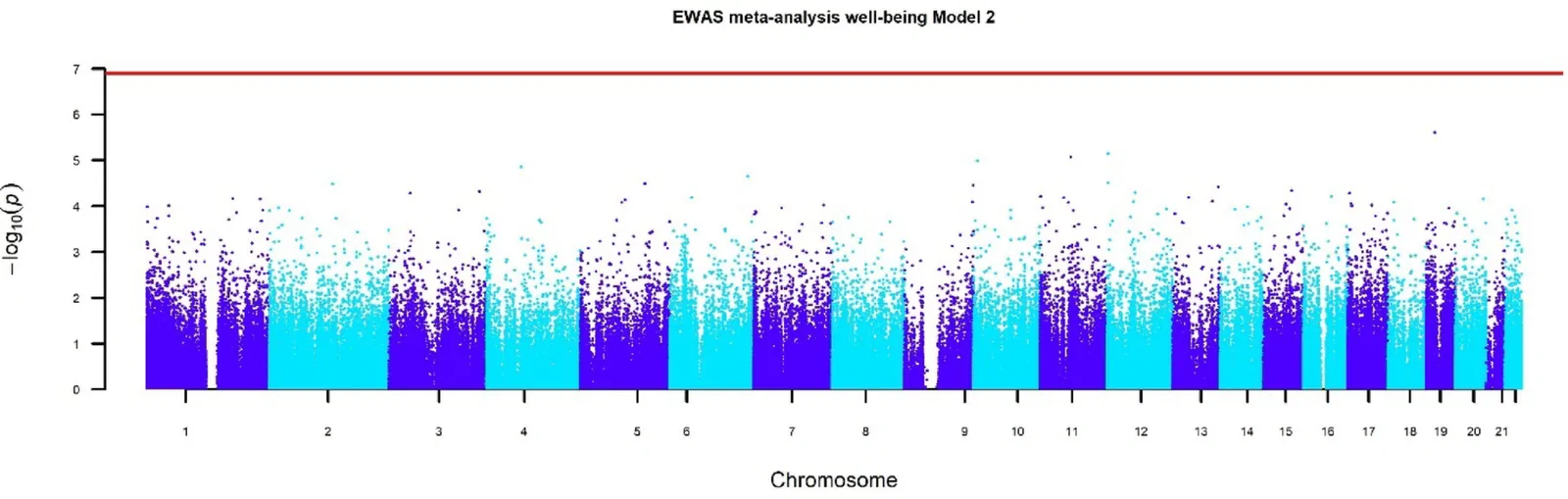
Manhattan plot for the EWAS meta-analysis with correction for smoking and BMI (model 2). The Manhattan plot shows the p-values for autosomal methylation sites from the EWAS meta-analysis of model 2, which included correction for smoking and BMI

**Fig. 3:**
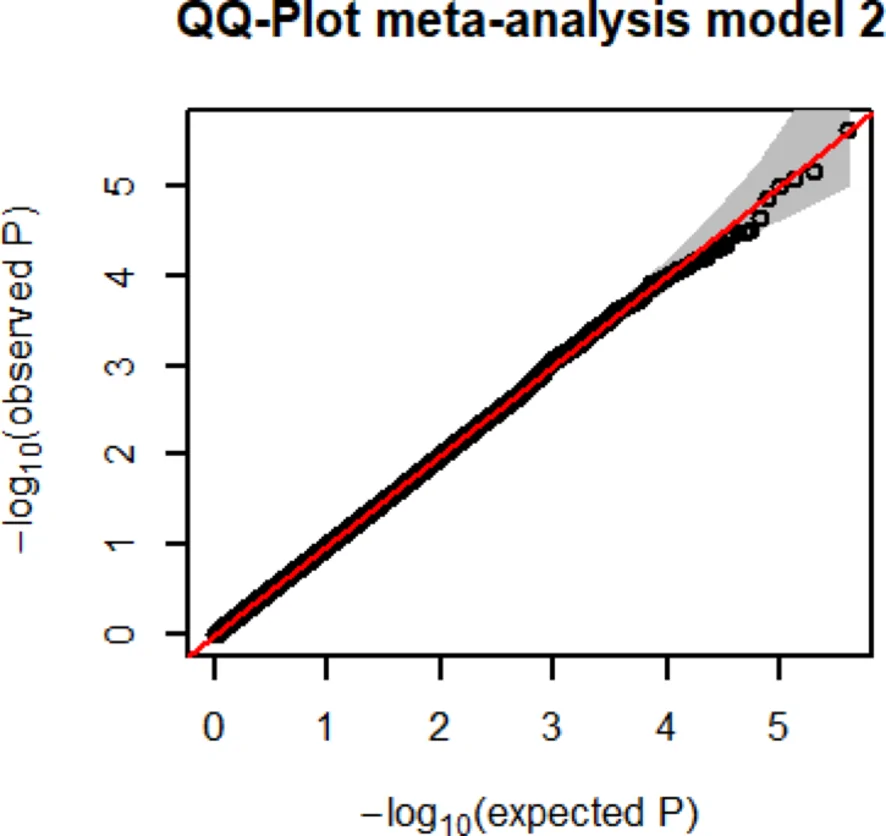
EWAS meta-analysis (model 2) QQ-Plot. QQ-plot from the EWAS meta-analysis of model 2, which included correction for smoking and BMI. The inflation factor (lambda) was 0.99

We performed two meta-analyses. The first EWAS meta-analysis (Model 1) included 13 studies with peripheral or cord blood DNA methylation data (N = 10,757) and was not corrected for smoking and BMI. The second EWAS meta-analysis (Model 2) consisted of 10 studies (N = 8,675) with peripheral blood DNA methylation data, as well as information about smoking data and BMI. The full summary statistics can be found in Supplementary Tables 6, 7. Results for the top 10 sites from Model 2 are shown in Table 3. No methylation sites were significantly associated with wellbeing in model 1 (supplementary Fig. 1 and supplementary Fig. 2) or model 2 (Figs. 2 and 3) after Bonferroni correction or at a false discovery rate of 5%**.** When inspecting heterogeneity statistics for both models, we observed that the top 10 sites (based on the outcomes from Model 2) showed mixed degrees of between-study heterogeneity (Model 1: mean I^2^ = 12.89%, range = 0–79.4%, Model 2: mean I^2^ = 12.99%, range = 0–81.9%), but only 2 CpGs (Model 1) and 1 CpG (Model 2), respectively, showed significant heterogeneity after Bonferroni correction (α = 1.2 × 10^–7^). We repeated the meta-analyses of model 1 after exclusion of one cohort with cord blood DNA methylation data (New N = 10,097). The correlation between effect sizes (Z-scores) from these two meta-analyses was high (*r* = 0.97). The meta-analysis without cord blood samples yielded no significant differentially methylated sites and showed a similar degree of heterogeneity (mean I^2^ = 13.19%, range = 0–81, significant heterogeneity at 1 CpG).

**Table 3:** Top-sites associated with wellbeing from the EWAS meta-analysis of model 2

CpG ID	CHR	Position^a^	Gene	N M1	Z-score M1	*P*-value M1	N M2	Z-score M2	*P*-value M2
cg13427283	19	17,411,666	*ABHD8*	10,754	3.763	0.000168	8660	4.708	2.51E− 06
cg05212836	12	2,986,733	*FOXM1;C12orf32*	10,498	− 4.826	1.39E−06	8636	− 4.488	7.19E−06
cg09370599	11	61,685,186	*RAB3IL1*	10,490	− 3.834	0.000126	8628	− 4.45	8.57E−06
cg23954465	10	8,078,038		10,752	− 4.737	2.17E−06	8657	− 4.41	1.03E−05
cg19045700	4	69,829,409		10,757	3.085	0.002032	8662	4.342	1.41E −05
cg03544657	6	158,487,564	*SYNJ2*	10,757	3.998	6.39E−05	8662	4.239	2.25E−05
cg15738421	12	2,986,597	*FOXM1;C12orf32*	10,749	− 3.841	0.000123	8656	− 4.163	3.14E− 05
cg02523014	5	130,688,743	*CDC42SE2;CDC42SE2*	10,753	2.54	0.01107	8658	4.153	3.28E−05
cg03450104	2	128,469,641	*WDR33*	10,753	3.887	0.000102	8658	4.151	3.31E−05
cg02076964	9	140,327,534	*NOXA1*	10,746	− 3.555	0.000379	8651	− 4.133	3.59E−05

^a^Genome build 37. M1, Model 1: correction for sex, age at blood sampling, white blood cell percentages (measured or imputed), and technical covariates; M2, Model 2 included the same predictors plus body-mass-index (BMI) and smoking status in adolescents and adults (current smoker, former smoker or never smoked)

### DNA methylation scores

We computed weighted DNA methylation scores in the NTR (peripheral blood, *Mean*_age_ = 37.77 years, *SD* = 12.99, *N* = 2506), based on the leave-one-out EWAS meta-analysis of model 2 without NTR. The best-performing MS that positively predicted wellbeing (which was the pruned score, with a *p*-value threshold of *P* < 1, i.e. based on all genome-wide pruned CpGs) explained 0.22% of the variation in wellbeing (b = 0.047, *p* = 0.0318), after adjusting for technical covariates, sex, age, standardized (age at blood sampling)^2^, cell counts, BMI, and smoking (Supplementary Table 8). Repeating the same analysis in a smaller subsample of NTR individuals who never smoked, the methylation score was no longer significantly predictive of wellbeing, but the effect size was fairly similar (*N* = 1437, *b* = 0.0357, *p* = 0.2357) suggesting that smoking does not drive the association between the methylation score and wellbeing.

### Combined performance of methylation and polygenic scores

Next, we tested whether the methylation score predicted wellbeing over and above a polygenic score (*N* = 2078). In a model that included the wellbeing polygenic score and the wellbeing methylation score, the wellbeing polygenic score was significant (*ß* = 0.0659, *R*^2^ = 0.4345%, *p* = 0.0046), but the methylation score was not significant (*ß* = 0.0256, *R*^2^ = 0.0653%, *p* = 0.2842, see Supplementary Table 9).

### Discordant twin pair analysis

We tested for differences in DNA methylation levels in monozygotic twin pairs discordant for wellbeing. There were no significant DNA methylation differences within the discordant pairs for the top 10 CpG sites from the EWAS meta-analysis without NTR (Supplementary Table 10). Five of the 10 top CpGs from the meta-analysis without NTR showed the same direction of effect in discordant pairs. The Q–Q plot for the comparison of discordant monozygotic twin pairs based on all genome-wide CpG sites is shown in Supplementary Fig. 3, illustrating that there was no inflation of test statistics and no genome-wide significant differences.

**Fig. 4:**
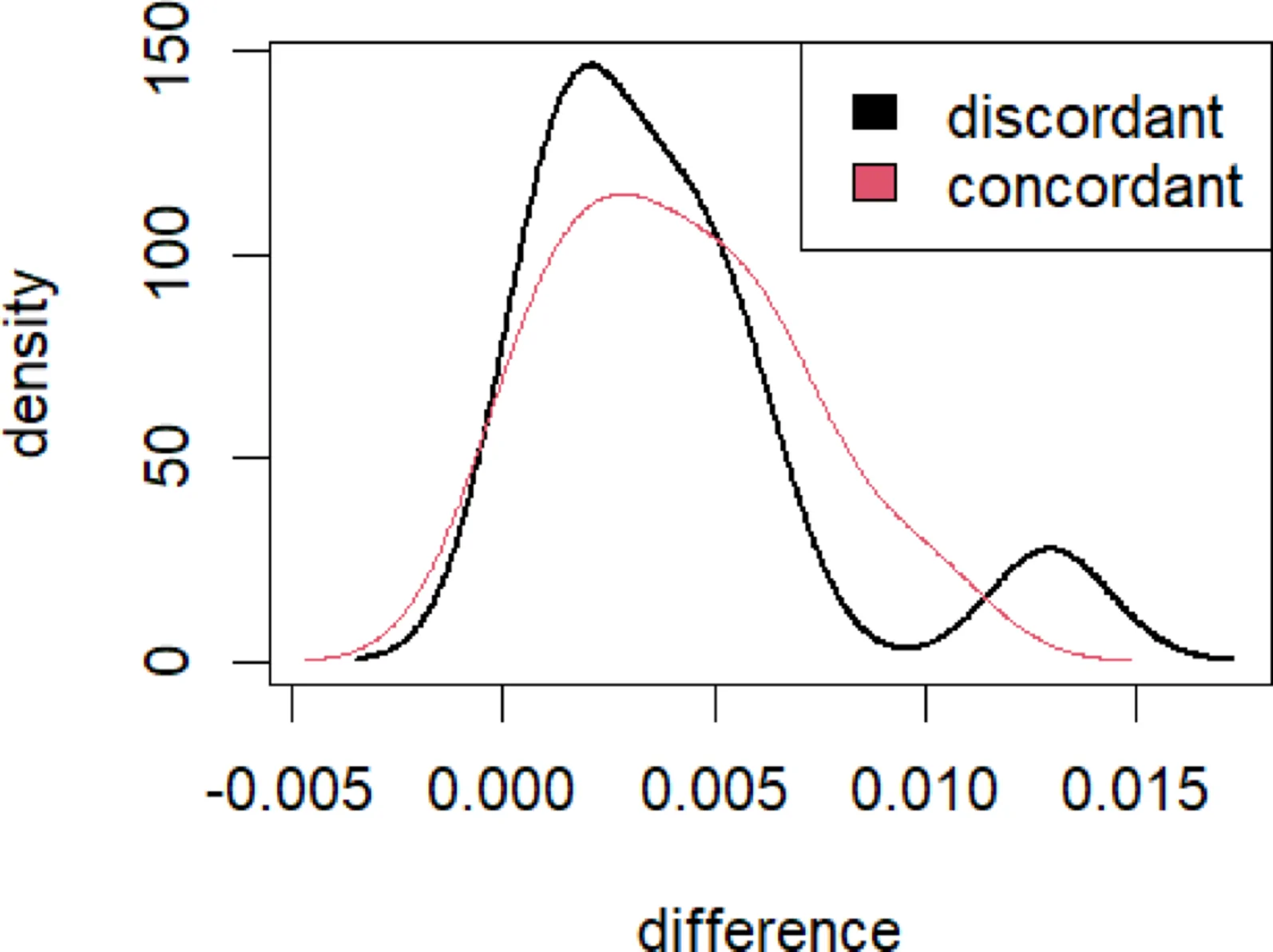
Density plot of the overall distribution of absolute within-pair DNA methylation differences in identical twin pairs based on the top 10 CpG sites from the leave-one-out EWAS meta-analysis of wellbeing (model 2 without NTR)

**Fig. 5:**
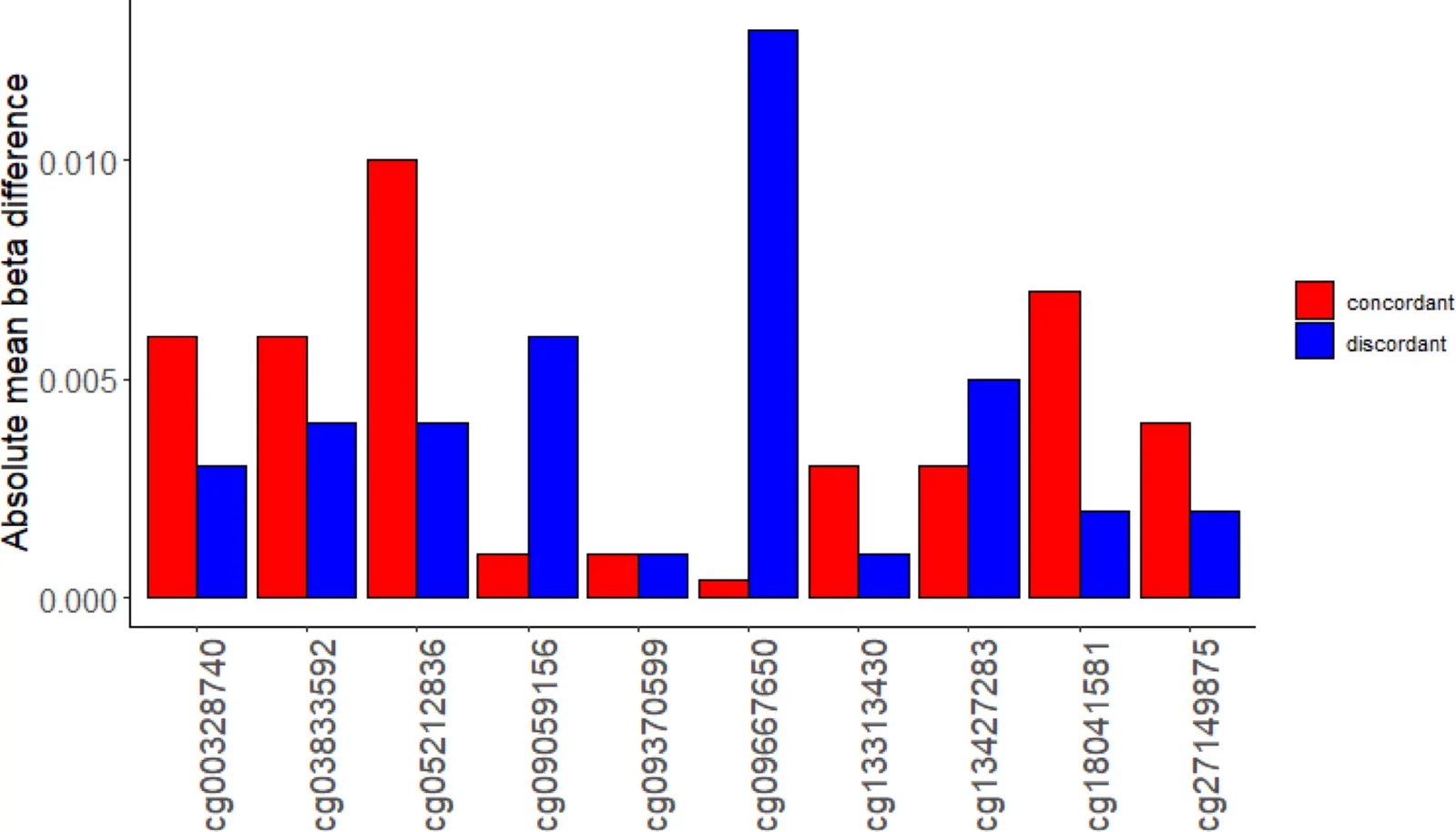
Absolute within-pair DNA methylation differences in wellbeing discordant and wellbeing concordant twin pairs for the top 10 CpGs from the leave-one-out EWAS meta-analysis (model 2 without NTR). For each of the top 10 CpG sites from the leave-one-out meta-analysis without NTR (model 2), the absolute average within-pair difference in DNA methylation is plotted

The distribution of the average within-pair absolute residual methylation difference for the top 10 CpGs did not differ significantly between discordant and concordant pairs (*p* = 0.99, Kolmogorov–Smirnov test) (see Figs. 4 and 5). Additionally, the DNA methylation scores of the discordant twins did not differ significantly between the wellbeing-high and wellbeing-low scoring twins from discordant monozygotic twin pairs (t = 0.12, *p* = 0.91).

## Discussion

This study is the first epigenome-wide association study meta-analysis of wellbeing. No genome-wide significant methylation sites were identified either without (N = 10,757) or with correction for BMI and smoking (N = 8,675). A weighted DNA methylation score based on all genome-wide CpGs from the summary statistics from our meta-analysis of wellbeing explained ~ 0.2% of the variance of wellbeing. Interestingly, the methylation score explained less variation in wellbeing and was no longer significantly predictive of wellbeing after adding the PGS based on the largest GWAS of the wellbeing spectrum to the model [4]. This suggests that the methylation score partially captures genetic variation in wellbeing that is also captured by the PGS [4]. In a secondary exploratory analysis, we used the unique characteristics of MZ twins to investigate site-specific methylation differences in monozygotic twin pairs discordant for wellbeing. This analysis did not identify significant methylation differences in wellbeing discordant monozygotic twin pairs.

Although our EWAS meta-analysis is among the largest EWAS meta-analyses to date with a sample size of ~ 11,000, it could be that the sample size is still too small to detect genome-wide significant CpGs for a complex behavioural trait such as wellbeing, for which small effect sizes might be expected. A blood-based EWAS meta-analysis of aggressive behavior [35], with a sample of over 14,000 individuals, also did not identify significant methylation sites after correcting for smoking and BMI. On the other hand, a recent EWAS meta-analysis of major depression with a sample size of 24,754 individuals identified 15 significant differentially methylated sites in blood [36]. We found that DNA methylation scores for wellbeing explained less variation (~ 0.2%) compared to methylation scores for, for instance, smoking (~ 60%), BMI (~ 10%), and educational attainment (~ 2%) [31, 37]; traits for which EWASs have been successful in identifying genome-wide significant CpGs with smaller sample sizes than ours. Although the explained variance of the wellbeing methylation score is small, it is in the same range as the variance explained by wellbeing polygenic scores based on DNA markers [2, 4].

We performed follow-up analyses in a unique group of MZ twin pairs discordant for wellbeing. As MZ twins share nearly 100% of their DNA sequence as well as much of their environment during childhood and adolescence, they form the ideal case–control group [34] as DNA methylation differences that will be found in a discordant EWAS model can be ascribed with more certainty to environmental influences and rule out most SNP-methylation effects. Epigenome-wide association studies using the discordant twin pair design have already been successfully applied for a number of behavioral traits, including schizophrenia and bipolar disorder, aggressive behavior, and autism [38–40]. However, we did not identify significant methylation differences within pairs of discordant twins in a replication analysis of the 10 top CpGs from the meta-analysis, with multiple testing correction for 10 tests. Since MZ pairs who are very discordant for wellbeing are rare, the sample size of this analysis was small and power was limited [41, 42]. According to a previously published power analysis [41], the power to detect DNA methylation level differences with 15 discordant pairs at epigenome-wide significance after multiple testing correction essentially approaches 0. This sample size only has fairly decent power (> = 70%) to detect within-pair DNA methylation differences of 10% or larger at nominal significance (alpha 0.05). Such large within-pair differences were not observed among top-ranking sites in the current analysis.

### Strengths and weaknesses

This is the largest EWAS of wellbeing to date, but it only included Illumina 450K array data, and future studies with a greater coverage of DNA methylation across the genome are warranted. The large sample size was achieved by including multiple measures of satisfaction with life and other wellbeing questionnaires including participants from multiple countries and all ages in a meta-analysis and analyzing DNA methylation data from blood. While this approach maximizes the power to detect common DNA methylation signatures of satisfaction with life and wellbeing, a limitation of this approach is that it will dilute age- and sex specific effects. Furthermore, the power to find statistically significant associations is reduced due to heterogeneity in phenotypic measurements and/or environmental background characteristics, as well as non-identical processing methods of methylation data. Our results indicate that larger EWA studies, or studies focusing on more homogenous measures of wellbeing, are needed to identify DNA methylation sites associated with wellbeing. In addition, other methods such as summary-data-based Mendelian Randomization [43] can be applied to summary statistics from large GWAS meta-analyses of wellbeing and summary statistics of methylation QTL analyses to increase our understanding of the functional consequences of SNP effects on wellbeing via DNA methylation, and to examine causal relationships between DNA methylation and wellbeing.

## Conclusions

In summary, in this first EWAS meta-analysis of wellbeing to date, we did not detect epigenome-wide significant associations between wellbeing and DNA methylation measured in blood while correcting for typical covariates including white blood cell counts, smoking and BMI. In addition, we found no significant DNA methylation differences in MZ twin pairs highly discordant for wellbeing. Larger meta-analyses and studies with more homogenous wellbeing measures are warranted.

## Supplementary Information

Below is the link to the electronic supplementary material.

Supplementary Material 1

Supplementary Material 2

Supplementary Material 3

Supplementary Material 4

Supplementary Material 5

Supplementary Material 6

Supplementary Material 7

## Data Availability

Genome-wide summary statistics from the EWAS meta-analysis are provided in Supplementary Tables 1 (Model 1) and 7 (Model 2). The SOP is available in supplementary information 1. EWAS linear model and gee scripts are provided in supplementary information 1. The meta-analysis script is provided in supplementary information 3.
