# Supplementary material for: Epigenome-wide association study meta-analysis of wellbeing
Source: Clin Epigenetics. 2026 Jul 14;18:181. doi: 10.1186/s13148-026-02202-0 (PMC13647890; doi:10.1186/s13148-026-02202-0)
Supplement: Supplementary file 3 [file 13148_2026_2202_MOESM3_ESM.docx]

**Supplementary information 3 - METAL meta-analysis code model 1.**

MARKER	cgid

WEIGHT	N

#ALLELE	EFFECT_ALLELE NON_EFFECT_ALLELE

EFFECT	Beta_SWB

STDERR	SE_SWB

PVAL	Pval_SWB

PROCESS Wellbeing_ALSPAC_happygenerally_Model1Ffilteredadjusted.txt

PROCESS Wellbeing_DTR_Happy2_Model1Ffilteredadjusted.txt

PROCESS Wellbeing_FTC2014_Model1Ffilteredadjusted.txt

PROCESS Wellbeing_KORAF4_Model1Ffilteredadjusted.txt

PROCESS Wellbeing_LBC1921_Model1Ffilteredadjusted.txt

PROCESS Wellbeing_LBC1936_Model1Ffilteredadjusted.txt

PROCESS Wellbeing_LLD_Model1Ffilteredadjusted.txt

PROCESS Wellbeing_NFBC1966_Model1Ffilteredadjusted.txt

PROCESS Wellbeing_NFBC1986_Model1Ffilteredadjusted.txt

PROCESS Wellbeing_QIMRB_Model1Ffilteredadjusted.txt

PROCESS Wellbeing_VatNormAge_lsi_Model1Ffilteredadjusted.txt

PROCESS Wellbeing_NTR_Model1Filteredadjusted_JVD.txt

\# Execute meta-analysis

ANALYZE HETEROGENEITY
