# Supplementary material for: Epigenome-wide association study meta-analysis of wellbeing
Source: Clin Epigenetics. 2026 Jul 14;18:181. doi: 10.1186/s13148-026-02202-0 (PMC13647890; doi:10.1186/s13148-026-02202-0)
Supplement: Supplementary file 4 [file 13148_2026_2202_MOESM4_ESM.docx]

**Supplementary figures**

**Supplementary Figure 1:** Manhattan plot for the EWAS meta-analysis of model 1

The Manhattan plot shows the p-values for autosomal methylation sites from the EWAS meta-analysis of model 1, without correction for smoking and BMI.

**Supplementary Figure 2:** QQ-Plot for the EWAS meta-analysis of model 1

QQ-plot from the EWAS meta-analysis of model 1, without correction for smoking and BMI. The inflation factor (lambda) was 1.03.

**Supplementary Figure 3:** QQ-Plot from the EWAS in wellbeing discordant twin pairs

QQ-Plot from the EWAS in wellbeing discordant monozygotic twin pairs. The inflation factor (lambda) was 1.01.
